# Lack of Intensity Control during an Exercise Program Is Related to a Limited Effect on Variables Responsible for Blood Pressure Regulation in Hypertensive Older Adults

**DOI:** 10.1155/2024/3128257

**Published:** 2024-06-27

**Authors:** Roberta Fernanda da Silva, Thaís Amanda Reia, André Mourão Jacomini, Anderson Bernadino da Silva, Henrique dos Santos Disessa, Henrique Luiz Monteiro, Anderson Saranz Zago

**Affiliations:** ^1^Department of Physical Education, Graduate Program in Movement Sciences, School of Sciences, Sao Paulo State University (Unesp), Bauru, Brazil; ^2^Department of Physical Education, Center for Noncommunicable Diseases, Aging and Exercise Studies (CEDEE), School of Sciences, Sao Paulo State University (Unesp), Bauru, Brazil

## Abstract

To compare the effect of an intensity-controlled exercise program (ICEP) and a nonintensity-controlled exercise program (non-ICEP) on the variables responsible for blood pressure regulation in hypertensive older adults. 95 hypertensive older adults (65.40 ± 7.48 years/22 males and 73 females) performed hemodynamic, functional fitness, and biochemical evaluations before and after 12 weeks of the multicomponent exercises which included walking, muscle strength, hydrogymnastics, Pilates, dynamic balance, agility, flexibility, and others. A significant improvement was observed in general functional fitness index (GFFI: *p* ≤ 0.000, *d* = 0.35), nitrite (NO_2_^−^: *p* ≤ 0.000, *d* = 0.49), systolic blood pressure (SBP: *p* ≤ 0.000, *d* = 0.65), diastolic blood pressure (DBP: *p* ≤ 0.013, *d* = 0.40), thiobarbituric acid reactive substances (TBARS: *p* ≤ 0.007, *d* = 0.78), activity of the endothelial superoxide dismutase enzyme (ecSOD: *p* ≤ 0.032, *d* = 0.41), double product (DP: *p* ≤ 0.015, *d* = 0.43), and waist-hip ratio (WHR: *p* ≤ 0.000, *d* = 0.44) for ICEP. Only GFFI (*p* ≤ 0.047, *d* = 0.12), TBARS (*p* ≤ 0.000, *d* = 0.77), SOD (*p* ≤ 0.025, *d* = 0.25), DP (*p* ≤ 0.046, *d* = 0.26), and BMI (*p* ≤ 0.018, *d* = 0.02) presented better results in non-ICEP. When the effect of the groups (controlled by age, BMI, and sex) was evaluated, an increase was observed in the NO_2_^−^, TBARS, and SOD and a reduction in the SBP and WHR variables in the ICEP group compared to the non-ICEP group. Twelve weeks of engagement in a controlled-intensity exercise program was enough to improve the level of functional fitness and variables regarding blood pressure regulation in hypertensive older adults. Conversely, physical exercise performed without intensity control was related to the limited effect on such variables.

## 1. Introduction

It is well established in the literature that regular physical exercise can benefit general health. It has been reported that both low-intensity and high-intensity exercises can promote protection against noncommunicable diseases, improve mental health, and prevent sarcopenia among other benefits, especially for the humoral mechanism of blood pressure (BP) regulation in older adults [1–3]. BP values are highly influenced by nitric oxide (NO) concentration, an important vasodilator, and by the activity of the renin-angiotensin-aldosterone system (RAAS), responsible for controlling vasoconstriction. The unbalance between these factors contributes to increased peripheral arterial resistance and, consequently, increased blood pressure values. The main factor suggested to promote this unbalance is oxidative stress. A high concentration of reactive oxygen species (ROS) promotes impairments in NO bioavailability and, consequently, impairments in blood pressure regulation [3–5]. Conversely, exercise is part of the nonpharmacological approach to treating hypertension (HT) [6]. Exercise stimulates an increase in NO concentration (vasodilation) [7], a reduction in RAAS activity through a decrease in the angiotensin-converting enzyme (ACE) activity (vasoconstriction) [8], and a reduction in oxidative stress [9] due to the increase in antioxidant enzymes, such as superoxide oxidase, catalase, and glutathione oxidase. Overall, exercise improves not only physical capacities [10], which promote greater independence and better quality of life in older adults [10–13], but also the humoral mechanism of BP regulation. Therefore, exercise is recommended to counteract the effects of aging and HT in hypertensive older adults [14].

Although the literature points out the benefits of staying physically active on blood pressure control pathways, these benefits are observed in intensity-controlled physical exercise programs (ICEPs) under supervision by specialized professionals. The general recommendation for exercise from the American Heart Association (AHA) is 150 minutes per week of moderate exercise or at least 75 minutes per week of systematically vigorous activity. Individuals can choose one intensity of physical activity or combine moderate and vigorous activities [15]. Overall, any exercise performed according to AHA guidelines should be enough to promote improvements in functional fitness, especially in some health variables, such as BP. Therefore, it is very common to find exercise programs for older adults being carried out without proper intensity control, leaving it up to the participants to decide what speed to walk or what load to use during resistance exercises, for example.

Studies indicate that nonintensity-controlled physical exercise programs (non-ICEPs) can encourage the practice of physical activities and socialization among older adults [16]. However, due to the lack of control over the physical exercise intensity, there are still doubts about the real benefits of this practice on BP control variables (vasodilator, vasoconstrictor, and oxidative stress). In addition, there is still little information in the literature on whether these programs provide benefits in the functional fitness index and BP control mechanisms in hypertensive older adults. Overall, it is not yet known whether physical exercise practiced without intensity control is sufficient to promote the same beneficial effect as physical exercises practiced with intensity control in hypertensive older adults. Therefore, the present study aimed to compare the effect of a physical exercise program with and without intensity control on the general functional fitness index, plasma nitrite responses, angiotensin-converting enzyme activity, oxidative stress profile, and blood pressure values in hypertensive older adults.

## 2. Methods

This longitudinal study included 95 hypertensive older adults (65.40 ± 7.48 years old) of both sexes (22 males and 73 females) from Bauru, São Paulo (Brazil). All volunteers participated in regular physical exercise programs linked to universities, gyms, or groups of older adults (details as follows). The exercises practiced included walking, muscle strength, hydrogymnastics, Pilates, or multicomponent exercises. Although these exercises have different characteristics, as aforementioned, they should all promote improvement in physical fitness and health.

All participants were invited to take part in this study (convenience sample) if they met the following inclusion criteria: aged between 60 and 80 years; having a medical diagnosis of HT and being under antihypertensive drug treatment; participating in a regular physical exercise program (duration of 60–75 minutes/session; a frequency of 2-3 sessions/week); nonsmokers; not regularly drinking alcohol (more than two daily doses); not presenting any medical, cardiological, orthopedic, and/or musculoskeletal condition that prevents participation in the respective physical exercise programs; and not having a diagnosis of any cardiovascular event, such as infarction and stroke, among other conditions. Antihypertensive drug treatment was not interrupted during the study. Moreover, participants who did not undergo all assessments in the pre- and postevaluations and did not comply with the 12 weeks of physical exercises proposed by the programs were excluded from the study. All participants were required to attend more than 75% of the sessions. Participants who specifically used antihypertensive drugs that act on the NO metabolism (nebivolol, carvedilol, and labetalol) and on the ACE activity (captopril and enalapril) were also excluded from the analysis.

This study was approved by the Research Ethics Committee of the Faculty of Sciences/UNESP-Bauru (approval no. 2.422.919/CAAE 78732617.2.0000.5398/C). All subjects provided their written informed consent form (WICF), after having all doubts clarified.

### 2.1. Sample Size

To determine the sample size, the coefficient of variation was initially calculated from the main variables (NO_2_^−^, ACE, SBP, and DBP). Due to the lack of data on this variable in the literature, information from the pilot study (40 subjects) was used. Thereby, the variable with the highest value/variability was adopted for the sample calculation (ECA: 50.59; NO_2_^−^: 27.21; SBP: 8.95; and DBP: 9.81).

**Figure 1:**
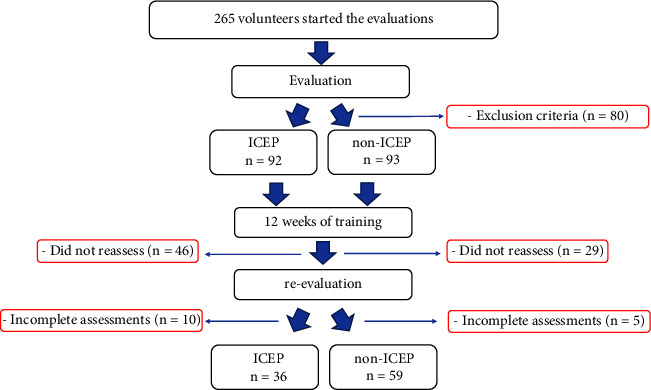
Participants selection process.

Therefore, the Miot' formula [17] was used to compare the paired samples according to the ACE activity quantitative variable (greater variability). It is important to emphasize that this study is part of a larger research project; therefore, 400 hypertensive older adults were invited to participate in this study based on the inclusion criteria (described above); however, only 265 older adults started the protocol. The final sample was made up of 95 participants who concluded all procedures of the protocol (Figure 1).

### 2.2. Characteristics of the Exercise Programs

To be selected for the study, each participant was required to be part of a regular physical exercise program, regardless of the modality (aerobic, resistance, combined, and multicomponent exercises, among others), and to be under the guidance of a physical education professional. These procedures were adopted because the specific aim of this study was not to evaluate the effect of any one type of physical exercise but to evaluate the effect of training status (TS) on the variables in question.

Initially, the principal researcher of this study identified whether the physical exercise programs were performed with the intensity controlled or not. All programs that included a controlled intensity of the exercise were classified as *intensity-controlled exercise programs (ICEPs).* Otherwise, all programs that did not control the intensity of the exercise were classified as *nonintensity-controlled exercise programs* (*non-*ICEPs).

The *ICEP* group performed structured and planned classes (warm-up, main part, and cool-down) which included aerobic activities, muscle strength, balance, flexibility, and other components of physical capacities (multicomponent exercises). The programs were performed with the following characteristics: 60–75 minutes/session, frequency of 2-3 sessions/week during 12 weeks; prescription of exercises according to the principles of training (multicomponent exercise) including 2 or more types of activities per day, and especially, with the intensity control and volume in an individualized way. Participants were instructed and encouraged to control the intensity of the activity using the subjective perception of effort scale, keeping the intensity between 11 (slightly tired) and 13 (tired) [18] and by heart rate reserve controlled by a heart rate monitor, keeping the intensity between 50 and 70% of RHR. Adjustment in the exercise intensity was perfomed (increase or decrease the workload) based on both parameters.

The *non-ICEP* group also had similar structured and planned class characteristics; however, this group had no control over the intensity of training in an individualized way. In general, both groups performed a similar program except for the control of the exercise intensity.

It should be noted that the intervention was administered by physical education professionals hired by the respective institutions. However, the researcher responsible for this study periodically monitored all activities through daily records with full access to the activities developed, frequency of participants in the classes, and intensity of the exercises to ensure that all criteria were being met.

### 2.3. Evaluations

All evaluations were performed before (baseline) and after (follow-up) 12 weeks of participation in the physical exercise programs. All evaluations were performed at the same time of day (between 7 am and 10 am) in July 2019 (before (baseline) participation in the physical exercise program) and at the reassessment (12-week follow-up) in November 2019.

Data collection was carried out in the “Noncommunicable Disease, Aging, and Physical Exercise Center (CEDEE)” from the School of Sciences, Sao Paulo State University (UNESP), Bauru, Brazil, in two separate visits for each moment (baseline and follow-up). During the first visit, participants answered a questionnaire and BP was measured according to the 7^th^ Brazilian Guideline of Arterial Hypertension [19]. A 12 h fasting blood collection was also performed to evaluate the biochemical variables. During the second visit, BP was measured again, and physical capacity tests were performed using the “American Alliance for Health, Physical Education, Recreation and Dance” (AAHPERD) battery test. The two visits were carried out on consecutive days and all older adults were instructed not to perform physical exercises in the 48 hours prior to the assessments.

It is important to emphasize that all evaluations were carried out by the CEDEE research members who received the same training to carry out all procedures and, therefore, maintained the same standardization during the evaluations.

- Questionnaires: participants answered a semistructured questionnaire, containing registration information, personal and family history related to their health condition, medical diagnoses, medications, and history and current habits of physical exercise.
- Anthropometric assessment: the classification of nutritional status and characterization of the participants were performed using the *body mass index* (BMI) and *waist-hip ratio* (WHR). BMI was calculated by dividing body mass in kilograms by height in meters squared [20]. The WHR was calculated by dividing waist circumference by hip circumference [20]. For the anthropometric assessment, a Tanita® (Model VM-080) digital weighing scale was used, which was adequately calibrated, with a capacity of 150 kg and a variation of 100 g and a stadiometer (SECA® 206), with a capacity of 220 cm, and an anthropometric tape, Sanny® TR4010 were also used. For the measurement, the participants remained standing, upright, and with the upper limbs close to the body. All participants were barefoot and wore light clothing during the evaluations.
- Hemodynamic evaluation: blood pressure (BP) was measured using the auscultatory method after participants rested for five minutes in a reserved and calm environment, sitting with their backs supported and their legs uncrossed, according to the recommendations of the 7^th^ Brazilian Guideline of Arterial Hypertension [19]. An aneroid sphygmomanometer device (Wan Med®, São Paulo, Brazil) and a stethoscope (Littmann®, Sumaré, Brazil) were used for BP measurement [19]. The double product (DP), an estimative of myocardial oxygen consumption and cardiac effort, was also calculated. This measurement was obtained by multiplying the heart rate (measured in bpm) by the systolic blood pressure (measured in mmHg). The normative double-product values vary on average from 6,000 at rest to 40,000 in strenuous exercise [21].
- Training status (TS) assessment: general functional fitness was assessed through the AAHPERD battery of tests, which involve the following capacities: coordination, flexibility, agility, muscular strength, and cardiovascular endurance. The complete description of each test was previously described [22, 23]. The result of each test was classified according to the normative values of the general functional fitness index (GFFI) developed by Zago and Gobbi [24], Benedetti et al. [25], and Benedetti et al. [26]. Each test received a score (percentile score), and the sum of these scores was used to calculate the individual GFFI. All assessments included in the AAHPERD battery showed good reliability and criterion validity for use in this age group. Test-retest reliability coefficients in the range of *r* = 0.80–0.99 have been reported for each assessment [22].
- Biochemical evaluation: blood collections were performed in the morning, between 7:00 am and 8:30 am, after 12 h fasting, following guidance to avoid foods rich in nitrates (beets, spinach, and arugula, among others) on the day before collection and not to practice physical exercises on the day of the test. At both moments, venous blood samples were collected through the antecubital vein by a professional specialized in this collection, using disposable materials. Sodium heparin tubes (9 ml) were used to determine the concentrations of nitrite (NO_2_^−^), a stable metabolite of NO, to quantify ACE activity, thiobarbituric acid reactive substances (TBARS), and the activity of the endothelial superoxide dismutase enzyme (ecSOD). The heparin tubes were centrifuged immediately after collection (4000 rpm for 5 minutes) for plasma separation and placed into a freezer at −80°C for future analysis.

Plasma nitrite concentrations were measured by reacting samples with Griess' reagent in microplates (96 wells) in an ELISA reader device. Total nitrite was analyzed by reacting 50 *μ*l of the sample with 50 *μ*l of the Griess reagent, and incubated at room temperature for 10 minutes on a plate shaker. Four different concentrations of nitrite (6 *μ*M, 12 *μ*M, 18 *μ*M, and 36 *μ*M) were used for the standard curve, which provided the equation of a straight line to perform the calculations. The absorbance reading of nitrite was performed at 545 nm [27].

The quantification of plasma ACE activity was performed by an automatic biochemical analyzer (A15 BioSystems S.A., Barcelona, CAT, Spain), using specific commercial kits (Angiotensin-Converting Enzyme, Code 12796, 50 ml/kit, BioSystems S.A., Barcelona).

The protocol adapted from Buege and Aust [28] was used for the lipoperoxidation analysis, enabling TBARS quantification. In a 15 ml conical falcon tube, 150 *µ*L of human plasma sample, 150 *µ*L of sodium decyl sulfate (SDS), 300 *µ*L of trichloroacetic acid (10%, w/v), and 500 *µ*L of thiobarbituric acid (0.67%, w/v) were mixed for reacting. The mixture was shaken and incubated for 30 minutes at 100°C and then cooled on ice. Subsequently, the mixture was centrifuged for 5 minutes at 4000 rpm at room temperature, and 200 *µ*L of the supernatant was removed from the centrifuged solution and added to the microplates (96 wells) for reading the absorbance at 535 nm in a spectrophotometer (Power Wave XS ELISA - BioTek instruments) [28].

To evaluate the antioxidant enzyme activity, a technique based on the inhibition of the superoxide radical reaction with pyrogallol was used. Pyrogallol is a compound that oxidizes itself with the pH variation, and this autoxidation generates superoxide radicals. The pyrogallol oxidation leads to the formation of a colored product, detected spectrophotometrically at 420 nm for 2 minutes. Since it is not possible to determine the concentration of the enzyme or its activity in the form of substrate consumed per unit of time, the quantification in relative units was used, in which one ecSOD unit is defined as the amount of enzyme required to inhibit the original detector oxidation rate by 50%. Thus, ecSOD activity was determined by measuring the rate of oxidized pyrogallol formation. For the reaction, 5 *µ*L of plasma sample, 980 *µ*L of tris-phosphate buffer (50 mmol/L; pH 8.2), 10 *µ*L of pyrogallol (24 mmol/L), and 5 *µ*L of catalase (30 *µ*mol/L) were used. Three different concentrations of ecSOD (6 *μ*M, 12 *μ*M, 18 *μ*M, and 36 *μ*M) were used for the preparation of the standard curve, which provided the equation of the straight line to perform the calculations [29].

### 2.4. Statistical Analysis

The Kolmogorov–Smirnov test was used to assess the data distribution considering the total sample (*n* = 95). In addition, the Shapiro–Wilk test was performed to evaluate the individualized distribution of each group. A repeated measure mixed two-way analysis of variance (ANOVA) and Levene test were conducted to compare intragroup and between-group data, with the type of program (ICEP and non-ICEP) as a between-subject factor, before and after 12 weeks of training as the intrasubject factor, and GFFI, NO_2_^−^, ACE activity, BP, TBARS, ecSOD, DP, BMI, and WHR as within-subject factors. The Friedman test (within groups) and Mann–Whitney *U* test (between groups) were used to analyze the nonparametric variables. In addition, univariate analysis of covariance (ANCOVA) was performed to confirm the results obtained using ANOVA, with age, BMI, and sex as covariates. Pairwise comparisons Bonferroni tests were also carried out for ANOVA and ANCOVA, with the GFFI, NO_2_^−^, ACE activity, BP, TBARS, ecSOD, DP, BMI, and WHR as dependent variables. To analyze the results, the Statistical Package for Social Sciences (SPSS, Chicago, IL, USA), version 22.0, was used, which presented the mean and SD for parametric analyses and the median and interquartile range for nonparametric analyzes. The level of significance was set at *p* ≤ 0.05. For pairwise comparison, we computed the effect size (Cohen's *d*), assumed as the standardized mean difference (*d* = (M1 − M2)/Ꝺ_pooled_). Threshold values were interpreted as *d* ≤ 0.3 (small), >0.3 (moderate), and >0.7 (large) [30].

## 3. Results

Table 1 shows the general characteristics of participants at baseline according to the physical exercise program (ICEP and non-ICEP) and the comparison between baseline and postintervention (follow-up). Age and sex did not present differences between groups (age 64.17 ± 7.72 vs 66.13 ± 7.29, *p* < 0.618; male/female: 7/29 vs 15/44, *p* < 0.502). Levene's test showed that the variances for NO_2_^−^ (*p* < 0.000), TBARS (*p* < 0.028), and SOD (*p* < 0.002) did not demonstrate homogeneity. Thereby, the median (interquartile range) of these variables in the baseline/F-B were as follows: ICEP group: NO_2_^−^: 0.09 (0.07–0.10)/0.130 (0.120–0.165); TBARS: 0.32 (0.28–0.34)/0.36 (0.31–0.38); and SOD: 2.20 (2.09–2.33)/2.29 (2.19–2.47) and the non-ICEP group: NO_2_^−^: 0.160 (0.140–0.210)/0.160 (0.140–0.200); TBARS: 0.36 (0.32–0.41)/0.31 (0.27–0.34); and SOD: 2.11 (1.90–2.33)/2.02 (1.88–2.29). Between-group Mann–Whitney analysis (ICEP vs. non-ICEP) demonstrated no significant differences in the majority of variables at baseline, except for DBP, TBARS, SOD, and NO_2_^−^. The non-ICEP group presented lower values of DBP (*p* ≤ 0.048, *d* = 0.01) and SOD (*U* = 682.500, *p* ≤ 0.038) and higher concentrations of TBARS (*U* = 506.000, *p* ≤ 0.000) and NO_2_^−^ (*U* = 154.000, *p* ≤ 0.000) compared to the ICEP group. At the postintervention moment (follow-up), the non-ICEP group presented higher values of NO_2_^−^ (*U* = 590.500, *p* ≤ 0.000) and SBP (*p* ≤ 0.025, *d* = −0.90) and lower values of GFFI (*p* ≤ 0.028, *d* = 0.05), TBARS (*p* ≤ 0.011, *d* = 0.13), and SOD (*p* ≤ 0.000, *d* = 0.68) compared with the ICEP group.

Table 1 also compares baseline and postintervention moments (follow-up) within each group. For the ICEP group, only ACE (*p* < 0.154) and BMI (*p* ≤ 0.882) variables did not show significant changes. All other variables presented better results after follow-up (GFFI: *p* ≤ 0.000, *d* = 0.35; NO_2_^−^: 0.130 (0.120–0.165); *X*^2^ = 24.029; *p* ≤ 0.000; SBP: *p* ≤ 0.000, *d* = 0.65; DBP: *p* ≤ 0.013, *d* = 0.40; TBARS: 0.36 (0.31–0.38); *X*^2^ = 9.966; *p* ≤ 0.002; SOD: 2.29 (2.19–2.47); *X*^2^ = 4.500; *p* ≤ 0.034; DP: *p* ≤ 0.015; *d* = 0.43 and WHR: *p* ≤ 0.000; *d* = 0.44). Conversely, for the non-ICEP group, NO_2_^−^ (*p* ≤ 0.785), ACE (*p* ≤ 0.217), SBP (*p* ≤ 0.081), DBP (*p* ≤ 0.926), SOD (*p* ≤ 0.102), and WHR (*p* ≤ 0.773) variables did not show significant changes. All other variables presented better results after follow-up (GFFI: *p* ≤ 0.047, *d* = 0.12; TBARS: 0.31 (0.27–0.34); *X*^2^ = 17.473; *p* ≤ 0.000; DP: *p* ≤ 0.046; *d* = 0.26 and BMI: *p* ≤ 0.018, *d* = 0.02).

An ANCOVA analysis was also performed to verify whether confounding factors could have affected the results obtained by ANOVA, such as age, BMI, and sex. In addition, a general univariate linear model analysis was performed to verify whether the ICEP and non-ICEP groups (independent variables) influenced the selected covariates. No influence between groups was detected for age (*p* ≤ 0.220), BMI (*p* ≤ 0.723), or sex (*p* ≤ 0.508) (Supplementary material (available here)).

To perform the ANCOVA analysis, a homogeneity test was carried out for each dependent variable. None of the variables presented statistical significance, indicating that the covariates did not affect the dependent variable in each group analyzed by this study; this result allows the ANCOVA to be performed. Overall, the ANCOVA showed that no covariate reached statistical significance when its effect was evaluated directly on the raw data of the dependent variables (Table 2).

The controlled means, adjusted by the covariates, are presented in Table 3 (age), Table 4 (BMI), and Table 5 (sex). It is possible to observe that the post hoc Bonferroni test demonstrated a relevant increase in the values of nitrite, TBARS, and ecSOD and a relevant reduction in the delta of SBP and WHR for the ICEP group compared with the non-ICEP group in both covariates. The DBP presented a reduction in the ICEP group only when sex was considered as a covariate (Table 5).

## 4. Discussion

As aforementioned, the purpose of this study was to compare the effect of intensity-controlled exercise program (ICEP) and nonintensity-controlled exercise program (non-ICEP) on the general functional fitness index, plasma nitrite responses, angiotensin-converting enzyme activity, oxidative stress profile, and blood pressure values of hypertensive older adults. The general results demonstrated that when the exercise program was carried out without intensity control (non-ICEP program), participants had some benefit, but to a limited extent. Conversely, when carried out with proper intensity control (ICEP program), the beneficial changes were much greater in variables responsible for blood pressure regulation in hypertensive older adults.

Both groups showed similar characteristics at baseline, except for oxidative stress (TBARS), DBP, ecSOD, and NO_2_^−^ concentration. Despite these differences, blood pressure values were within the normal range for both groups. In addition, it is important to note that the control of antihypertensive drugs was not interrupted during the study. These results suggest that the baseline variables related to oxidative stress did not affect the variables related to BP regulation.

When both groups were submitted to the physical training program, the ICEP group showed significant improvements in blood pressure regulation variables, while the non-ICEP group showed limited improvements (Table 1). In the ICEP group, 12 weeks of physical training generated positive changes in GFFI and almost in all substances that participated in blood pressure regulation (increase in NO_2_^−^, ecSOD, and TBARS and reduction in DP and WHR values). SBP and DBP were also reduced in this group. In addition, in the non-ICEP, only the GFFI, DP, and WHR values showed positive variations between baseline and follow-up, characterized by a small increase in the TS level, lower myocardial energy expenditure, and lower cardiovascular risk. Although significant differences were observed in the prooxidant substances (TBARS), no difference was observed in the antioxidant substances (ecSOD). In agreement with these results, some studies have shown that physical exercise reduces oxidative damage, improves the antioxidant defense system, and increases the resistance of organs and tissues against the deleterious action of free radicals [3–5, 31]. For example, Jacomini and collaborators evaluated the influence of functional fitness and oxidative capacity on the concentration of nitric oxide associated with hemodynamic control in older adult women (65.73 ± 6.14 years). Participants were separated according to training status (TS1, very weak and weak; TS2, regular; and TS3, good and very good). Blood plasma samples were also used to evaluate the prooxidant and antioxidant activity and nitrite and nitrate concentrations. The general results of this study showed that good TS levels were related to lower levels of lipoperoxidation and protein damage, higher antioxidant levels, and higher nitrite and nitrate concentrations. This combination may be responsible for the lower BP levels in individuals with better TS [31]. In addition, the review study by Polidoro and colleagues sets out to summarize current experimental, clinical, and epidemiological knowledge on known associations and potential links between oxidative stress and physical activity/exercise during aging. They concluded that this relationship plays a preventive and therapeutic role in antioxidant supplementation in older adults who practice exercise, but there is a substantial need for more studies on this topic [5]. In fact, the results of the present study suggest that a physical exercise program performed with no intensity control did not promote major changes in blood pressure regulation variables. This finding reinforces the need for hypertensive older adults to maintain a better training status and to receive adequate guidance/prescription for physical exercises, as in the ICEP group.

The means adjusted by the covariates, presented in Table 3 (age), Table 4 (BMI), and Table 5 (sex), showed a relevant increase in the delta of nitrite, TBARS, and SOD and a relevant reduction in the SBP and WHR for the ICEP group compared with the non-ICEP group for all covariates (Tables 3, 4, 5). In addition, DBP decreased in the ICEP group only when sex was considered as a covariate (Table 5). It is well established in the literature that physical exercise itself promotes several benefits to the human body [13, 32–35]. The Study by Saco-Ledo and collaborators proposed to evaluate the effects of physical training on BP in patients with hypertension based on evidence from randomized clinical trials. The benefits of exercise on all BP measures were significant for patients, and only aerobic exercise provided significant benefits. Aerobic exercise is an effective adjunctive treatment for reducing BP in medicated patients with hypertension [35]. The study by Myers, Kokkinos, and Nyelin provides an overview of the effects of physical activity and greater physical fitness on metabolic syndrome, along with a discussion of the mechanisms underlying the benefits of being fitter or more physically active in preventing and treatment of metabolic syndrome [33]. From another perspective, the study by Silva et al. [13] aimed to use accelerometry to examine the relationship between sedentary time, light physical activity, and moderate to vigorous physical activity with the physical fitness of older adults. The results reinforce the importance of promoting the practice of moderate to vigorous physical activity among older adults, thus allowing the maintenance or improvement of physical fitness [13]. Although the non-ICEP group did not present significant differences in the current study, the literature indicates that the regular practice of physical activity, even without intensity control, has benefits for the level of physical activity and quality of life when compared with sedentary older adults [36]. Silva et al. [36] compared the levels of physical activity and quality of life between older adults who practice regular physical exercise and sedentary older adults. The authors verified the association between the level of physical activity and quality of life in the groups. They evaluated 50 older adults (70.24 ± 8.8 years), divided into two groups: sedentary (G1, *n* = 25) and those who practice regular physical exercise (G2, *n* = 25). According to the results, older adults who practice physical exercise and sedentary older adults had a good level of physical activity. However, older adult people who exercise regularly had a higher level of physical activity, explaining the better quality of life in this group [36]. In the current study, the physical exercise program without intensity control did not seem to be sufficient to optimize the positive changes in the variables of blood pressure regulation. Conversely, the ICEP program was essential for blood pressure regulation in hypertensive older adults. These results are supported by Rogers et al. [37], who investigated whether self-reported physical activity intensity (sedentary, light, moderate, or vigorous) could significantly reduce frailty trajectories in older adults. As a result, light physical activity was insufficient to significantly delay the progression of frailty compared to sedentary older adults. Conversely, moderate physical activity reduced the progression of frailty in some age groups (particularly 65 years and older), while vigorous activity significantly reduced the trajectory of frailty progression in all older adult age groups [37].

In general, the findings of the current study demonstrate that hypertensive older adults should participate in an exercise program with intensity control to optimize as much as possible the variables related to blood pressure regulation. This result may explain why some studies did not show reductions in blood pressure values after a physical exercise program. It is possible that the exercise intensity was not sufficient to provide these benefits or that the initial level of training status was weak. Another important finding, but not explored by this study, concerns the cutoff point in the GFFI score to promote these positive results. In the ICEP group, almost all variables showed improved results after 12 weeks of the exercise program, and the GFFI variation was approximately 32 points. Conversely, in the non-ICEP program, this score was only 12.5 points. Although an increase of approximately 32 GFFI points in the ICEP group appears to be sufficient to obtain such benefits, this needs further investigation.

The findings of the current study are relevant for public health managers, as the data indicate the effectiveness of intensity-controlled exercise programs (ICEPs) associated with pharmacological treatment to improve variables related to blood pressure regulation. Overall, adequate control of exercise intensity could be extremely important to facilitate positive changes in variables responsible for blood pressure regulation in hypertensive older adults. To the best of our knowledge, this is one of the few studies to explore the effects of ICEP and non-ICEP programs in older adult hypertensive patients.

## 5. Conclusion

It is already well known that the practice of physical exercise brings health benefits, however, for such benefits to be significant, it is important that the exercise must be carried out following some characteristics, such as intensity control of exercise. Our results demonstrated that physical exercise performed without intensity control was related to a limited effect especially on variables related to blood pressure regulation. Conversely, twelve weeks of engagement in a controlled-intensity exercise program was enough to improve the level of functional fitness (GFFI) and variables related to blood pressure regulation in hypertensive older adults such as NO_2_^−^, TBARS, ecSOD, DP, and WHR.

Although it has been common to find exercise programs for older adults being carried out without proper intensity control, it is important to emphasize that the control of intensity during the exercise sessions can optimize the results, as demonstrated by this study.

## 6. Study Limitations

The time of previous practice of physical exercise among participants was different and the sedentary group was not included in the analysis. Overall, the literature already points out that the practice of regular physical exercises brings benefits to older adults' health in comparison to sedentary groups. Therefore, the authors did not emphasize the no-exercise group in the experimental design of this study but rather emphasized the effect of controlling exercise intensity on the physical fitness level and blood pressure control variables. Although this could interfere with the initial data, baseline results demonstrated homogeneous characteristics among participants, especially in the GFFI (*p* < 0.214 in the ICEP and non-ICEP comparison). Nutritional status was not controlled by this study; however, participants were advised to maintain the same dietary pattern as before the beginning of the procedures. Also, it is important to highlight that although the number of participants per group was different, both groups included the minimum number of participants established by the sample size calculation (at least 16 participants per group).

## Figures and Tables

**Table 1:** Dependent variables of the ICEP and non-ICEP groups at baseline and follow-up.

	ICEP group (*n* = 36)			Non-ICEP group (*n* = 59)			ICEP × non-ICEP	
	Baseline	Follow-up	F-B	Baseline	Follow-up	F-B	Baseline	Follow-up
			*P* value			*P* value	*P* value	*P* value
Age (years)	64.17 ± 7.72	—	—	66.13 ± 7.29	—	—	0.618	—
Male/female	7/29	—	—	15/44	—	—	0.502	—
GFFI (points)	268.95 ± 94.26	301.01 ± 87.65	0.000^*∗*^	242.07 ± 105.72	254.65 ± 104.31	0.047^*∗*^	0.214	0.028^†^
°NO_2_^−^ (nM)	0.11 ± 0.10	0.15 ± 0.07	0.000^*∗*^	0.18 ± 0.06	0.18 ± 0.06	0.785	0.000^†^	0.000^†^
ACE (nm/min/ml)	36.83 ± 26.10	32.57 ± 21.16	0.154	43.48 ± 24.22	40.64 ± 19.39	0.217	0.214	0.063
SBP (mmHg)	129.92 ± 13.14	120.67 ± 15.23	0.000^*∗*^	130.01 ± 13.07	127.31 ± 12.86	0.081	0.972	0.025^†^
DBP (mmHg)	80.14 ± 8.18	76.83 ± 8.40	0.013^*∗*^	76.77 ± 7.77	76.87 ± 7.94	0.926	0.048^†^	0.983
°TBARS (nM/mg)	0.32 ± 0.04	0.36 ± 0.06	0.002^*∗*^	0.37 ± 0.06	0.32 ± 0.07	0.000^*∗*^	0.000^†^	0.000^†^
°SOD (U SOD/mg protein)	2.25 ± 0.23	2.34 ± 0.21	0.034^*∗*^	2.13 ± 0.30	2.06 ± 0.27	0.102	0.038^†^	0.000^†^
Double product	9587.52 ± 1429.39	8968.00 ± 1433.27	0.015^*∗*^	9875.80 ± 1555.98	9477.24 ± 1486.94	0.046^*∗*^	0.406	0.133
BMI (kg/m^2^)	30.44 ± 4.77	30.42 ± 4.64	0.882	30.06 ± 5.22	29.81 ± 5.31	0.018^*∗*^	0.738	0.588
WHR	0.89 ± 0.07	0.86 ± 0.07	0.000^*∗*^	0.88 ± 0.09	0.87 ± 0.09	0.773	0.468	0.334

*Note.* F-B, follow-up to baseline; ICEP, intensity-controlled exercise program; non-ICEP, nonintensity-controlled exercise program; GFFI, general functional fitness index; NO_2_^−^, nitrite concentration; ACE, angiotensin-converting enzyme; SBP, systolic blood pressure; DBP, diastolic blood pressure; TBARS, thiobarbituric acid reactive substances; SOD, superoxide dismutase enzyme; BMI, body mass index; WHR, waist-hip ratio. Statistically significant when *p* < 0.05. ^*∗*^Within groups (baseline to follow-up). ^†^Between groups (baseline to baseline or follow-up to follow-up). °Nonparametric analysis (Mann–Whitney *U* test or Friedman test) after Levene's homogeneity test.

**Table 2:** Effect of age, BMI, and sex covariations on dependent variables.

Variable	Age				BMI				Sex			
	df	*F*	*p*	*R* ^2^	df	*F*	*p*	*R* ^2^	df	*F*	*p*	*R* ^2^
GFFI (points)	1.91	0.684	0.410	0.023	1.85	0.002	0.968	0.012	1.92	0.934	0.336	0.027
NO_2_^−^ (nM)	1.91	0.306	0.581	0.126	1.85	3.302	0.073	0.138	1.92	0.227	0.635	0.132
ACE activity (nm/min/ml)	1.90	1.080	0.302	0.009	1.84	0.344	0.559	0.014	1.91	0.001	0.981	0.020
SBP (mmHg)	1.91	0.005	0.945	0.044	1.85	1.012	0.317	0.039	1.92	0.424	0.517	0.053
DBP (mmHg)	1.91	0.107	0.745	0.020	1.85	0.399	0.530	0.009	1.92	0.225	0.636	0.025
TBARS (nM/mg)	1.87	2.902	0.092	0.219	1.81	0.034	0.854	0.233	1.88	0.367	0.546	0.209
SOD (U SOD/mg protein)	1.86	0.007	0.933	0.071	1.80	0.011	0.933	0.086	1.87	0.025	0.847	0.078
Double product	1.77	0.656	0.420	0.006	1.71	0.457	0.501	0.020	1.78	0.082	0.775	0.018
BMI (kg/m^2^)	1.78	0.366	0.547	0.012	1.79	0.415	0.521	0.007	1.79	0.177	0.675	0.004
WHR	1.78	3.762	0.056	0.159	1.79	0.258	0.613	0.107	1.79	0.110	0.741	0.105

df, degrees of freedom; *F*, mean squares ratio; *R*^2^, adjusted *R*-squared; GFFI, general functional fitness index; NO_2_^−^, nitrite concentration; ACE, angiotensin-converting enzyme; SBP, systolic blood pressure; DBP, diastolic blood pressure; TBARS, thiobarbituric acid reactive substances; SOD, superoxide dismutase enzyme; BMI, body mass index; WHR, waist-hip ratio. ^*∗*^Statistically significant when *p* < 0.05.

**Table 3:** Bonferroni post hoc analysis using the age covariate under the mean delta of the ICEP and non-ICEP groups.

Variable	Group	Mean	Mean difference (ICEP and non-ICEP)	Std. error	*p*	95% CI	
						Lower	Upper
GFFI (points)	ICEP	31.290	18.302	10.420	0.082	−2.396	39.001
	Non-ICEP	12.987				−39.001	2.396

NO_2_^−^ (nM)	ICEP	0.042	0.40	0.011	0.001^*∗*^	0.019	0.061
	Non-ICEP	0.002				−0.061	−0.019

ACE activity (nm/min/ml)	ICEP	−4.498	−1.840	3.928	0.632	−9.446	5.765
	Non-ICEP	−2.657				−5.765	9.446

SBP (mmHg)	ICEP	−9.071	−6.375	2.559	0.015^*∗*^	−11.458	−1.282
	Non-ICEP	−2.697				1.292	11.458

DBP (mmHg)	ICEP	−3.127	−3.196	1.696	0.063	−6.565	0.174
	Non-ICEP	0.069				−1.174	6.565

TBARS (nM/mg)	ICEP	0.039	0.091	0.018	0.001^*∗*^	0.055	0.126
	Non-ICEP	−0.051				−0.126	−0.055

SOD (U SOD/mg protein)	ICEP	0.086	0.157	0.054	0.005^*∗*^	0.050	0.264
	Non-ICEP	−0.071				−0.264	−0.050

Double product	ICEP	−715.126	−330.821	322.169	0.308	−972.342	310.700
	Non-ICEP	−384.305				−310.700	972.342

BMI	ICEP	0.011	0.264	0.158	0.100	−0.051	0.579
	Non-ICEP	−0.253				−0.579	0.051

WHR	ICEP	−0.034	−0.031	0.009	0.001^*∗*^	−0.049	−0.013
	Non-ICEP	−0.002				0.013	0.049

Std. error, standard error; GFFI, general functional fitness index; NO_2_^−^, nitrite concentration; ACE, angiotensin-converting enzyme; SBP, systolic blood pressure; DBP, diastolic blood pressure; TBARS, thiobarbituric acid reactive substances; SOD, superoxide dismutase enzyme; BMI, body mass index; WHR, waist-hip ratio. ^*∗*^Statistically significant when *p* < 0.05.

**Table 4:** Bonferroni post hoc analysis using the BMI covariate under the mean delta of the ICEP and non-ICEP groups.

Variable	Group	Mean	Mean difference (ICEP and non-ICEP)	Std. error	*p*	95% CI	
						Lower	Upper
GFFI (points)	ICEP	32.074	19.009	10.855	0.084	−2.573	40.592
	Non-ICEP	13.064				−40.592	2.573

NO_2_^−^ (nM)	ICEP	0.043	0.037	0.010	0.001^*∗*^	0.017	0.058
	Non-ICEP	0.006				−0.058	−0.017

ACE activity (nm/min/ml)	ICEP	−4.345	−2.696	3.916	0.493	−10.483	5.092
	Non-ICEP	−1.649				−5.092	10.483

SBP (mmHg)	ICEP	−9.194	−5.195	2.483	0.039^*∗*^	−10.132	−0.257
	Non-ICEP	−3.999				0.257	10.132

DBP (mmHg)	ICEP	−3.282	−2.548	1.678	0.133	−5.884	0.789
	Non-ICEP	−0.734				−0.789	5.884

TBARS (nM/mg)	ICEP	0.040	0.097	0.019	0.001^*∗*^	0.060	0.134
	Non-ICEP	−0.057				−0.134	−0.060

SOD (U SOD/mg protein)	ICEP	0.092	0.172	0.055	0.003^*∗*^	0.062	0.282
	Non-ICEP	−0.080				−0.282	−0.062

Double product	ICEP	−637.237	−160.408	343.113	0.642	−844.557	523.740
	Non-ICEP	−476.829				−523.740	844.567

BMI	ICEP	−0.015	0.236	0.157	0.137	−0.077	0.549
	Non-ICEP	−0.252				−0.549	0.077

WHR	ICEP	−0.033	−0.031	0.009	0.001^*∗*^	−0.049	−0.013
	Non-ICEP	−0.002				0.013	0.049

Std. error, standard error; GFFI, general functional fitness index; NO_2_^−^, nitrite concentration; ACE, angiotensin-converting enzyme; SBP, systolic blood pressure; DBP, diastolic blood pressure; TBARS, thiobarbituric acid reactive substances; SOD, superoxide dismutase enzyme; BMI, body mass index; WHR, waist-hip ratio. ^*∗*^Statistically significant when *p* < 0.05.

**Table 5:** Bonferroni post hoc analysis using the sex covariate under the mean delta of the ICEP and non-ICEP groups.

Variable	Group	Mean	Mean difference (ICEP and non-ICEP)	Std. error	*p*	95% CI	
						Lower	Upper
GFFI (points)	ICEP	31.643	18.808	10.200	0.068	−1.449	39.066
	Non-ICEP	12.835				−39.066	1.449

NO_2_^−^ (nM)	ICEP	0.043	0.042	0.010	0.001^*∗*^	0.021	0.062
	Non-ICEP	0.001				−0.062	−0.021

ACE activity (nm/min/ml)	ICEP	−4.257	−1.414	3.783	0.710	−8.927	6.100
	Non-ICEP	−2.844				−6.100	8.927

SBP (mmHg)	ICEP	−9.320	−6.657	2.507	0.009^*∗*^	−11.636	−1.678
	Non-ICEP	−2.663				1.678	−11.636

DBP (mmHg)	ICEP	−3.339	−3.455	1.665	0.041^*∗*^	−6.762	−0.148
	Non-ICEP	0.115				0.148	6.762

TBARS (nM/mg)	ICEP	0.040	0.090	0.018	0.001^*∗*^	0.054	0.126
	Non-ICEP	−0.050				−0.126	−0.054

SOD (U SOD/mg protein)	ICEP	0.091	0.162	0.053	0.003^*∗*^	0.057	0.267
	Non-ICEP	−0.071				−0.267	−0.057

Double product	ICEP	−609.355	−204.496	324.805	0.531	−851.132	442.141
	Non-ICEP	−404.860				−442.141	851.132

BMI	ICEP	−0.015	0.238	0.158	0.136	−0.076	0.552
	Non-ICEP	−0.252				−0.552	0.076

WHR	ICEP	−0.033	−0.031	0.009	0.001^*∗*^	−0.049	−0.013
	Non-ICEP	−0.002				0.013	0.049

Std. error, standard error; GFFI, general functional fitness index; NO_2_^−^, nitrite concentration; ACE, angiotensin-converting enzyme; SBP, systolic blood pressure; DBP, diastolic blood pressure; TBARS, thiobarbituric acid reactive substances; SOD, superoxide dismutase enzyme; BMI, body mass index; WHR, waist-hip ratio. ^*∗*^Statistically significant when *p* < 0.05.

## Data Availability

All data generated or analyzed during this study are included in this manuscript.

## Supplementary Materials

Supplementary Materials An ANCOVA analysis was also performed to verify whether confounding factors could have affected the results obtained by ANOVA, such as age, BMI, and sex. In addition, a general univariate linear model analysis was performed to verify whether the ICEP and non-ICEP groups (independent variables) influenced the selected covariates. No influence between groups was detected for age (*p* ≤ 0.220), BMI (*p* ≤ 0.723), or sex (*p* ≤ 0.508).
