# Supplementary material for: Lack of Intensity Control during an Exercise Program Is Related to a Limited Effect on Variables Responsible for Blood Pressure Regulation in Hypertensive Older Adults
Source: J Aging Res. 2024 Jun 27;2024:3128257. doi: 10.1155/2024/3128257 (PMC11223912; doi:10.1155/2024/3128257)
Supplement: Supplementary Materials — An ANCOVA analysis was also performed to verify whether confounding factors could have affected the results obtained by ANOVA, such as age, BMI, and sex. In addition, a general univariate linear model analysis was performed to verify whether the ICEP and non-ICEP groups (independent variables) influenced the selected covariates. No influence between groups was detected for age (p ≤ 0.220), BMI (p ≤ 0.723), or sex (p ≤ 0.508). [file 3128257.f1.docx]

**SUPPLEMENTARY MATERIAL**

**Supplementary material 1**

*Effect Of The Covariate-Adjusted Group On The Dependent Variables*

| **Variable** | **Age** | | | | | **BMI** | | | | **Sex** | | | | |
| --- | --- | --- | --- | --- | --- | --- | --- | --- | --- | --- | --- | --- | --- | --- |
|  | df | F | p | R² | df | | F | p | R² | | df | F | p | R² |
| **GFFI (Points)** | 1.91 | 3.085 | 0.082 | 0.023 | 1.85 | | 3.067 | 0.084 | 0.012 | | 1.92 | 3.400 | 0.068 | 0.027 |
| **NO_2_^-^ (nM)** | 1.91 | 14.335 | 0.001 | 0.126 | 1.85 | | 13.165 | 0.001 | 0.138 | | 1.92 | 16.271 | 0.001 | 0.132 |
| **ACE Activity (nm/min/ml)** | 1.90 | 0.231 | 0.632 | 0.009 | 1.84 | | 0.474 | 0.493 | 0.014 | | 1.91 | 0.140 | 0.710 | 0.020 |
| **SBP (mmHg)** | 1.91 | 6.205 | 0.015 | 0.044 | 1.85 | | 4.376 | 0.039 | 0.039 | | 1.92 | 7.051 | 0.009 | 0.053 |
| **DBP (mmHg)** | 1.91 | 3.548 | 0.063 | 0.020 | 1.85 | | 2.305 | 0.133 | 0.009 | | 1.92 | 4.306 | 0.041 | 0.025 |
| **TBARS (nM/mg)** | 1.87 | 25.463 | 0.001 | 0.219 | 1.81 | | 27.143 | 0.001 | 0.233 | | 1.88 | 25.077 | 0.001 | 0.209 |
| **SOD (U SOD/ mg protein)** | 1.86 | 8.456 | 0.005 | 0.071 | 1.80 | | 9.687 | 0.003 | 0.086 | | 1.87 | 9.471 | 0.003 | 0.078 |
| **DOUBLE PRODUCT** | 1.77 | 1.054 | 0.308 | 0.006 | 1.71 | | 0.219 | 0.642 | 0.020 | | 1.78 | 0.396 | 0.531 | 0.018 |
| **BMI (kg/m^2^)** | 1.78 | 2.777 | 0.100 | 0.012 | 1.79 | | 2.261 | 0.137 | 0.007 | | 1.79 | 2.270 | 0.136 | 0.004 |
| **WHR** | 1.78 | 12.277 | 0.001 | 0.159 | 1.79 | | 11.558 | 0.001 | 0.107 | | 1.79 | 11.529 | 0.001 | 0.105 |

*Note.* df: Degrees of freedom; F: Mean squares ratio; R²: Adjusted R squared. GFFI: General Functional Fitness Index; NO_2_^-^: nitrite concentration; ACE: Angiotensin-converting enzyme; SBP: systolic blood pressure; DBP: diastolic blood pressure; TBARS: thiobarbituric acid reactive substances; SOD: Superoxide dismutase Enzyme; BMI: body mass index; WHR: waist-hip ratio. * Statistically significant when p <0.05.
